# Leptin signaling maintains autonomic stability during severe influenza infection in mice

**DOI:** 10.1172/JCI182550

**Published:** 2024-10-31

**Authors:** Andrés R. Muñoz-Rojas, Adam C. Wang, Lisa E. Pomeranz, Elizabeth L. Reizis, Heather W. Stout-Delgado, Ileana C. Miranda, Krishnan Rajagopalan, Tadiwanashe Gwatiringa, Roger R. Fan, Ahmad A. Huda, Neha Maskey, Roseline P. Olumuyide, Aryan S. Patel, Jeffrey M. Friedman, Diane Mathis, Kartik N. Rajagopalan

**Affiliations:** 1 Department of Immunology, Harvard Medical School, Boston, Massachusetts, USA.; 2 Laboratory of Molecular Genetics, Howard Hughes Medical Institute, Rockefeller University, New York, New York, USA.; 3 Department of Medicine, Division of Pulmonary and Critical Care Medicine, Weill Cornell Medicine, New York, New York, USA.; 4 Laboratory of Comparative Pathology, Weill Cornell Medicine, Memorial Sloan Kettering Cancer Center, and The Rockefeller University, New York, New York, USA.; 5 Department of Internal Medicine, Division of Pulmonary and Critical Care, UT Southwestern Medical Center, Dallas, Texas, USA.

**Keywords:** Endocrinology, Infectious disease, Influenza, Leptin

## To the Editor:

Mice with deficient hypothalamic leptin signaling have increased susceptibility to influenza (1). To better understand the role of leptin in the response to influenza infection, we infected leptin-deficient ob/ob mice with influenza and found that they had a high mortality rate, which was completely prevented by leptin.

**Figure 1:**
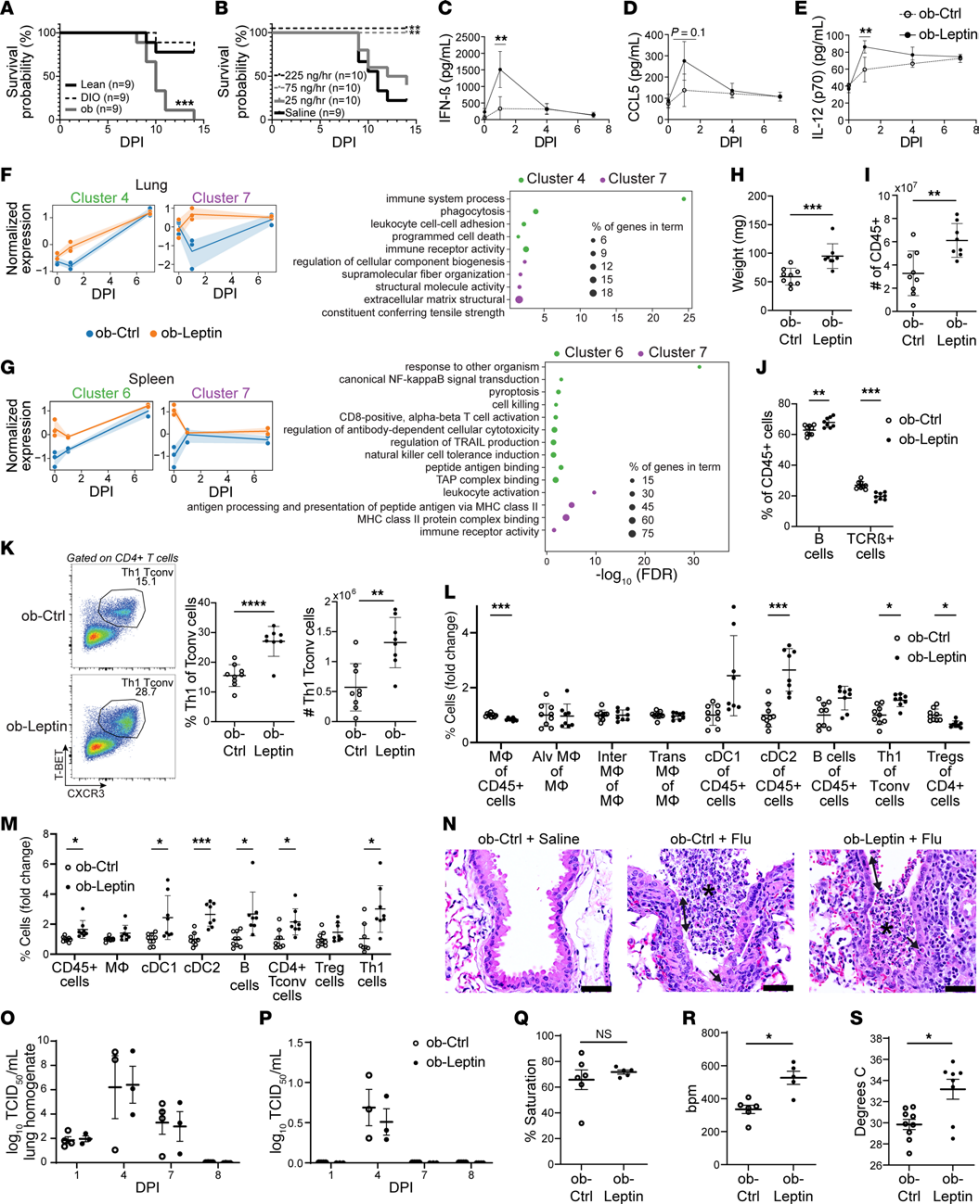
Leptin supplementation maintains autonomic stability during severe influenza infection. (**A**) Kaplan-Meier curve of influenza A–infected WT lean and DIO mice and *ob/ob* mice and (**B**) influenza A–infected *ob*/*ob* mice supplemented with leptin. (**C**–**E**) Serum levels of (**C**) IFN-β (**D**) CCL5, and (**E**) IL-12. (**F** and **G**) Whole-tissue RNA-Seq of (**F**) lung and (**G**) spleen. Average normalized expression values for the chosen gene clusters across time (left), and functional enrichment analysis for each cluster (right). (**H**) Weight, (**I**) number of immunocytes, and (**J**) proportion of cell types in spleens 8 days after infection. (**K**) Representative flow cytometry plots (left) and quantification (right) of Th1 cells in spleens. (**L** and **M**) Immunophenotyping of lungs 8 days after infection, showing the fold change in (**L**) proportions and (**M**) numbers of different cell types. (**N**) H&E staining of lungs. Normal, uninfected tissue is shown (left). Seven days after infection, both Ob-ctrl–infected (middle) and ob-leptin–infected (right) tissues showed attenuation of the epithelium (black double-headed arrows), necrotic epithelial cells (black arrows), peribronchiolar infiltrates of immune cells (white double-headed arrows), and luminal debris (black asterisk). Flu, influenza. Original magnification, ×20. Scale bars: 40 μm. (**O** and **P**) Viral titers of (**O**) lung homogenates (**P**) and serum. (**Q**) Oxygen saturation (**R**) heart rate, and (**S**) core temperature of *ob*/*ob* mice 7 days after infection. OB, *ob/ob*; DPI, days post infection; Mθ, macrophages; Alv Mθ, alveolar macrophages; Inter Mθ, interstitial macrophages; Trans Mθ, transitional macrophages; TCID_50_, 50% tissue culture infectious dose; bpm, beats per minute. *P < .05, **P < .01, ***P < .005, ****P < .001, by 2-tailed Student’s *t* test. Data represent the mean ± SEM.

While diet-induced obese (DIO) mice have fasting hyperglycemia and weight comparable to those of ob/ob mice (Supplemental Figure 1, A and B; supplemental material available online with this article; https://doi.org/10.1172/JCI182550DS1), inoculation of 100 PFU influenza A/PR/8/34 caused 100% mortality in ob/ob mice (Figure 1A) but not DIO mice, indicating that leptin deficiency rather than adiposity and metabolic dysregulation was the major contributor to increased mortality in ob/ob mice. Chronic leptin supplementation completely reversed mortality (Figure 1B), even though these mice ate less food and lost more weight (Supplemental Figure 1, C and D), while acute leptin supplementation prior to infection had no rescue effect (Supplemental Figure 1, H–J).

We measured serum levels of cytokines in control ob/ob mice (ob-ctrl) and mice supplemented with 75 ng/h leptin (ob-leptin) after infection. Certain antiviral cytokines and chemokines were elevated in ob-leptin mice one day after infection and normalized by day four (Figure 1, C–E); other cytokines showed no difference (Supplemental Figure 2A).

Whole-tissue sequencing (RNA-Seq), differential gene expression analysis, and hierarchical clustering on RNA from the lungs and spleens of ob-ctrl and ob-leptin mice revealed several differences (Supplemental Figure 3). Gene set enrichment analysis (GSEA) showed two gene clusters in lung that were expressed at higher levels in ob-leptin mice and were enriched for “immunologic activation” and “extracellular matrix” pathways (Figure 1F). Two gene clusters in spleen had higher expression levels in ob-leptin mice and were enriched for “T cell activation,” “immunologic activation,” and “antigen-processing” pathways (Figure 1G). These results suggest that leptin increased immunologic activation early in infection, which normalized by day seven.

We performed immunophenotyping eight days after infection and found a marked increase in both the spleen size and number of CD45+ cells in ob-leptin mice (Figure 1, H and I), including an increase in the proportion of B cells and a concomitant decrease in the proportion of T cell receptor β (TCRβ+) cells (Figure 1J). Th1 T cells in the spleen were increased in ob-leptin mice (Figure 1K), indicating an improved Th1 antiviral systemic response. Immunophenotyping of the lungs showed that the proportions of conventional DCs (cDC2s) and Th1 cells were increased in the ob-leptin group, while the proportions of macrophages and Tregs in the lungs were decreased (Figure 1L). These differences were accounted for by increases in the number of DCs, T conventional (Tconv) cells, Th1 T cells, B cells, and total CD45+ cells in ob-leptin mice (Figure 1M). These analyses are consistent with an elevated Th1 antiviral response in ob-leptin mice.

Despite the marked difference in mortality, there was no difference in lung (Figure 1N), trachea (Supplemental Figure 4C), heterogeneity of lung injury (Supplemental Figure 4D), or oxygen saturation (Figure 1Q) between the groups. Despite a better Th1 antiviral response in ob-leptin mice, ob-ctrl mice had no difficulty clearing the virus, and lung and serum viral titers were similar between the two groups (Figure 1, O and P). In addition, blockade of type I IFN signaling in ob-leptin mice did not reverse survival (Supplemental Figure 2B), indicating that leptin’s effects might not be due to augmentation of immune function. Instead, leptin treatment prevented profound bradycardia and hypothermia that was seen after viral infection in ob-ctrl mice (Figure 1, R and S).

Leptin deficiency caused a profoundly increased susceptibility to influenza infection, which was completely reversed with chronic physiologic leptin replacement prior to infection. Although ob-ctrl mice had deficient Th1 responses, they showed viral clearance that was equal to that of the treated group. However, untreated animals developed profound bradycardia and hypothermia prior to death, which were prevented by leptin treatment. Leptin is known to stimulate hypothalamic neurons to modulate sympathetic nerve fibers and control heart rate and temperature (2). Our findings raise the possibility that leptin signaling is implicated in preserving autonomic function after severe viral infection.

## Supplementary Material

Supplemental data

Supporting data values

## Version 1 10/31/2024

In-Press Preview

## Version 2 01/02/2025

Electronic publication
