# Supplementary material for: Leptin signaling maintains autonomic stability during severe influenza infection in mice
Source: J Clin Invest. 2024 Oct 31;135(1):e182550. doi: 10.1172/JCI182550 (PMC11684820; doi:10.1172/JCI182550)
Supplement: Supplemental data [file jci-135-182550-s154.pdf]

Sex as a biological variable

Male mice were used for the experiments described in this study due to availability. However, our unpublished data show that female wild-type and leptin-deficient *ob/ob* mice respectively display 0 - 20% mortality and 80 - 100% mortality, similar to the male cohorts described in the main text of this study. Female *ob/ob* mice also exhibit impaired autonomic function during influenza infection as measured by significantly decreased body temperature prior to death. We therefore expect our finding that leptin promotes survival during viral infection via autonomic, non-immune mechanisms to be relevant to both male and female sexes.

Statistics

All statistical analyses were performed using GraphPad Prism software, except for the Python NumPy package, which was used to perform summary statistics on the oximeter data. Log-rank test was performed to compare Kaplan-Meier survival curves. Unpaired Student's t-test was performed to compare data from two groups. Holm-Sidak method was used to correct for multiple comparisons.

Study approval

All animal procedures were conducted in accordance with the National Institutes of Health Guide for the Care and Use of Laboratory Animals and were approved by the Institutional Animal Care and Use Committee of The Rockefeller University and UT Southwestern Medical Center (protocol numbers 23026-H & 20013 for The Rockefeller University and 2023-103537 for UT Southwestern Medical Center).

Data availability

RNA-sequencing data has been deposited to the National Center for Biotechnology Information Gene Expression Omnibus and has been assigned GEO GSE274568. A single XLS file containing values for all data points in the graphs for all figures in the manuscript is reported in the Supporting Data Values file.

Acknowledgments

We gratefully acknowledge: H. Hoffman and C. Rice for influenza reagents and protocols; K. Seddu, A. Baysoy, J. Lee, I. Magill, and the Broad Genomics Platform for RNA-seq; the HMS Immunology Flow Core; and P. Cohen, J. Elmquist, and L. Terada for discussions. This work was supported by NIH T32GM152349 (to A.C.W.), NHLBI T32HL105323 (to K.N.R.), a Rockefeller Clinical Scholars KL2 (to K.N.R.), Stony Wold-Herbert Fund Fellowship Grant (to K.N.R.) and grants from the JPB Foundation (to J.M.F. and D.M.). The Laboratory of Comparative Pathology at MSKCC is supported by the NCI Cancer Center Support Grant P30 CA008748.

Author contributions

K.N.R., A.R.M-R, J.M.F. and D.M. conceived and/or designed the study. K.N.R., A.C.W., A.R.M-R., and L.E.P. designed and performed all experiments except for viral titration and histopathology. H.W.S-D. performed viral titration. I.C.M. supervised histopathological sample preparation and interpreted data. K.R. assisted with analysis of pulse oximetry data. E.L.R., T.G., A.A.H., N.M., R.P.O., R.R.F., and A.S.P. assisted with experiments and acquired data. K.N.R., A.R.M-R., and A.C.W. wrote the manuscript. K.N.R., J.M.F., and D.M. edited the manuscript and supervised the study. A.R.M-R. is the first-listed co-first author because he was involved in the study's conception.

Methods

Animals. Male C57BL/6J mice at 8 weeks of age (The Jackson Laboratory, #000664) were group housed and placed on either standard rodent chow diet (20% protein & 4.5% fat, LabDiet® 5053) or high-fat diet (20% protein & 60% fat, Research Diets D12492) for 16 weeks prior to experimentation. Male *ob/ob* mice were either purchased (The Jackson Laboratory, #000632) at 10 weeks of age or obtained through in-house breeding and maintained in standard conditions until 14-18 weeks of age for experimentation. Because of the nature of the study, weight loss, reduction in body temperature, or movement were not used as criteria for euthanasia.

Viral propagation. Mouse-adapted influenza A/PR/8/34 virus (IAV PR8, a gift from Charlie Rice at Rockefeller University) was propagated and titered using MDCK (ATCC CCL-34) cells as previously described (3, 4). Briefly, monolayers of MDCK cells at 90% confluency were infected with IAV PR8 at a multiplicity of infection of 0.001 and incubated until cells exhibited near 100% cytopathic effect (approximately 3 days). Supernatant from infected cells was collected, cleared of cell debris (3,200*g* at 4°C for 10 min) and aliquots stored at -80°C. Clarified cell supernatant was used for all infections.

Influenza viral infection. All mice were anesthetized with isoflurane (5% for induction and 2% for maintenance) prior to intranasal inoculation with 100 PFU PR8 in 50 µl of PBS. Daily food intake, body weight, and mortality was recorded for 14 days after PR8 administration.

Leptin treatment. For chronic leptin administration in *ob/ob* mice, 42-day ALZET mini-osmotic pumps (DURECT Corporation, Model 2006) were pre-incubated at 37 degrees C for 60 hours and then were subcutaneously implanted with to deliver recombinant mouse leptin (R&D Systems, 498-OB) at the specified doses in sterile 0.9% normal saline (Medline, DYND40540) for 3 weeks starting 1 week prior to infection. For acute leptin administration, the same procedure was followed except that pumps were incubated prior to implantation for 12 hours and then implanted two days prior to infection so that part of the equilibration would occur in the mouse and leptin infusion would begin at the time of infection.

In vivo IFNAR blockade. *ob/ob* mice receiving either saline or leptin via osmotic pump implants were treated with IFNAR neutralizing antibody (clone MAR1-5A3; BioXCell) or Anti-Mouse IgG1 Isotype Control (clone MOPC-21; BioXCell) as previously described (5). Briefly, 500 ug of anti-IFNAR or IgG isotype control antibody was administered intraperitoneally (i.p.) per mouse on days -1 and 0 post-PR8 infection. On days 2, 4, and 6 post-infection, each mouse was treated with 250ug of either anti-IFNAR or IgG isotype control antibody.

Plasma cytokine measurements. Plasma was collected and snap frozen. The Mouse Anti-Virus Legendplex (BioLegend) was used to measure plasma cytokines according to the manufacturer’s instructions. The beads were run on a FACSymphony analyzer (BD Biosciences).

Whole-tissue RNAseq library preparation, sequencing and data processing. Total RNA was isolated using the RNAeasy Lipid Tissue Mini Kit (Qiagen). 2ng of RNA were diluted in 5μl of Buffer TCL (Qiagen). Libraries were constructed, sequenced, and processed as outlined in the Immgen protocol (https://www.immgen.org/img/Protocols/ImmGenULI_RNAseq_methods.pdf), and as previously described (6). Read counts were normalized and analyzed for differential gene expression using the DEseq2 package (7). Differentially expressed genes were clustered using hierarchical clustering with the scipy package in python using the ward linkage and euclidean distance. GSEA was performed using gprofiler2 in R[3]. Plotting and downstream analysis was performed in python.

Isolation of immunocytes from lung and spleen.

Lung: Isolation was performed as previously described (8). For isolation of lung immunoctyes, the lungs were excised, minced and digested at 37ºC for 40 min in Roswell Park Memorial Institute (RPMI) medium containing 2% fetal calf serum (FCS), collagenase IV (0.5mg/mL, Gibco) and DNAse I (100μg/mL) in a shaking water bath. Digested tissues were passed through a 70μm cell strainer and washed in RPMI/FCS. Red blood cells were lysed using ammonium-chloride-potassium (ACK) buffer, washed again, and resuspended for antibody staining.

Spleen: Spleens were mechanically disassociated, filtered through a 40μm cell strainer, and washed with Dulbecco's Modified Eagle's Medium (DMEM) + 2% FCS.

Flow cytometry. Cell suspensions were stained for flow cytometry with the following antibodies: anti-CD4, -CD8a, -CD11b, -CD11c,- CD19, -CXCR3, -CD64, -CD45, -TCRgd, -T-bet,

MHC-II, all from BioLegend, anti-Foxp3, -ST2 from eBioscience, and anti-TCRb from BD Biosciences. The antibody clone and catalog numbers are summarized in the table below. Cell surface staining was performed for 20 min on ice in DMEM/FCS. Viability was measured with the LIVE/DEAD Fixable Blue Dead Cell Stain Kit (Invitrogen) according to the manufacturer's instructions. Fixing, permeabilizing and intracellular staining was performed with the Foxp3/ Transcription Factor Staining Buffer Set (eBioscience). Cells were acquired in a Cytek Aurora spectral cytometer (Cytek Biosciences).

**Antibody**	**Manufacturer**	**Clone**	**Catalog #**
anti-CD4	BioLegend	RM4-5	100550
anti-CD8a	BioLegend	53-6.7	100710
anti-CD11b	BioLegend	M1/70	101226
anti-CD11c	BioLegend	N418	117335
anti-CD19	BioLegend	6D5	115528
anti-CXCR3	BioLegend	CXCR3-173	126527
anti-CD64	BioLegend	X54-5/7.1	139309
anti-CD45	BioLegend	30-F11	103140
anti-TCRɣδ	BioLegend	GL3	118117
anti-T-bet	BioLegend	4B10	644810
anti-MHC-II	BioLegend	M5/114.15.2	107614
anti-Foxp3	eBioscience	FJK-16s	53-5773-82
anti-ST2	eBioscience	RMST2-2	25-9335-82
anti-TCRb	BD Biosciences	H57-597	612821

Necropsy and histopathology. Eight days after viral inoculation, were submitted for postmortem evaluation by a board-certified comparative pathologist at the Laboratory for Comparative Pathology of The Rockefeller University, Memorial Sloan Kettering Cancer Center, and Weill Cornell Medicine, New York, NY, USA. Mice were euthanized with carbon dioxide overdose and a complete macroscopic examination was performed.

All tissues were fixed in 10% neutral-buffered formalin for 72 hours, followed by decalcification of bones in a formic acid solution (Surgipath Decalcifier I, Leica Biosystems). Formalin-fixed tissues were then routinely processed in ethanol and xylene and embedded in paraffin in a tissue processor (Leica ASP6025, Leica Biosystems). Paraffin blocks were sectioned at 5 μm and stained with hematoxylin and eosin (H&E). The following tissues were processed and examined histologically: white and brown adipose tissues, heart, thymus, lungs, liver, gallbladder, kidneys, pancreas, stomach, duodenum, jejunum, ileum, cecum, colon, lymph nodes (submandibular, mesenteric), salivary glands, skin (trunk and head), urinary bladder, uterus, cervix, vagina, ovaries, oviducts, adrenal glands, spleen, thyroid gland, esophagus, trachea, spinal cord, vertebrae, sternum, femur, tibia, stifle join, skeletal muscle, nerves, skull, nasal cavity, oral cavity, teeth, ears, eyes, pituitary gland, and brain.

Viral load measurement. $TCID_{50}$ was calculated using the Viral ToxGlo Assay (Promega, Madison WI). Briefly, lung homogenate and serum was diluted in 3.16-fold serial dilutions and plated for 72 hours on >80% confluent MDCK cells. Upon visualization of cytopathic effect, ATP detection reagent was added and luminescence was measured. Values were calculated by plotting net relative luminescence units (RLU) values after subtracting average blank wells against viral dilution. The TCID50 value is the reciprocal of the dilution that produced a 50% decline in ATP levels compared to untreated controls.

Measurement of body temperature. Core temperature was measured in awake mice using a probe (Rodent Rectal Temperature Probe, WPI RET-3) inserted 2 cm into the rectum while animals were placed under light, brief manual restraint.

Pulse oximetry and heart rate measurement. Arterial oxygen saturation and heart rate were monitored in influenza-infected mice using the MouseOx® Plus Mouse & Rat Pulse *Oximeter (Starr Life Sciences)*. The oximeter collected animal biometric data at approximately 60 millisecond intervals over a 3-hour period of monitoring using collar sensors that did not restrict movement around their home cages. During recording, animals were provided with moist diet gels (Clear H_2O, DietGel® 93M) that allowed them free access to nutrition and hydration. Data generated were analyzed by Python script using the open-source packages “NumPy” for summary statistics and “pandas” for cleaning and organizing the data. The data is read-in from a comma separated values file provided by the software. It is initially parsed by removing all invalid measurements with a non-zero error code. Next, an index was created for every subject and the valid data was grouped by subject. The subject-grouped data was used to derive average, median, and standard deviation for subjects’ heart rate and arterial oxygen levels.

3. Balish AL, Katz JM, and Klimov AI. Influenza: propagation, quantification, and storage. *Curr Protoc Microbiol.* 2013;Chapter 15:15G 1 1-G 1 24.
4. Eisfeld AJ, Neumann G, and Kawaoka Y. Influenza A virus isolation, culture and identification. *Nat Protoc.* 2014;9(11):2663-81.
5. Macal M, Jo Y, Dallari S, Chang AY, Dai J, Swaminathan S, et al. Self-Renewal and Toll-like Receptor Signaling Sustain Exhausted Plasmacytoid Dendritic Cells during Chronic Viral Infection. *Immunity.* 2018;48(4):730-44 e5.
6. Mann AO, Hanna BS, Munoz-Rojas AR, Sandrock I, Prinz I, Benoist C, et al. IL-17A-producing gammadeltaT cells promote muscle regeneration in a microbiota-dependent manner. *J Exp Med.* 2022;219(5).
7. Love MI, Huber W, and Anders S. Moderated estimation of fold change and dispersion for RNA-seq data with DESeq2. *Genome Biol.* 2014;15(12):550.
8. Munoz-Rojas AR, Wang G, Benoist C, and Mathis D. Adipose-tissue regulatory T cells are a consortium of subtypes that evolves with age and diet. *Proc Natl Acad Sci U S A.* 2024;121(4):e2320602121.

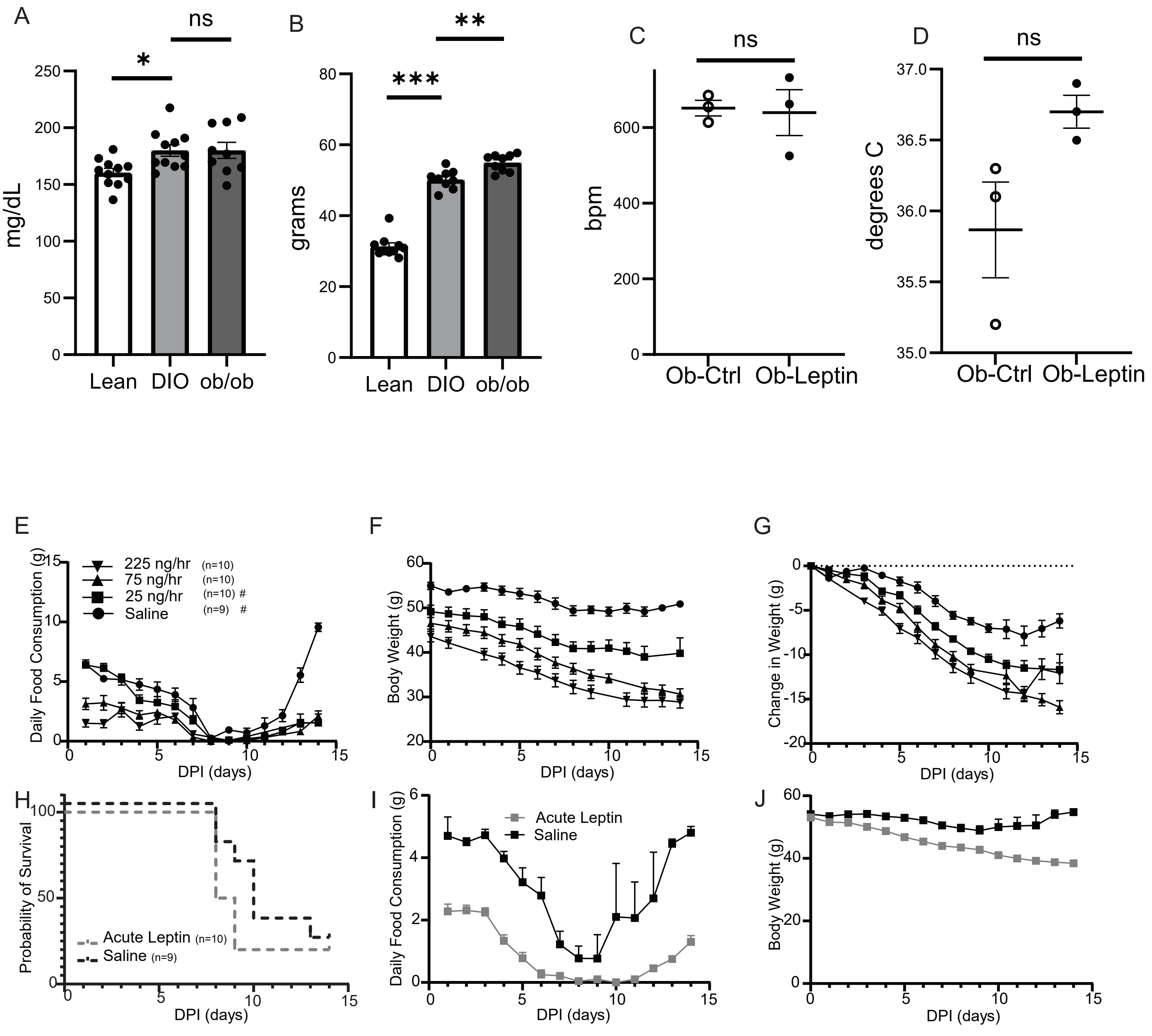

Figure S1. Glycemia, weight, food intake, heart rate, core temperature, and weight loss of lean, DIO, and control ob/ob mice and ob/ob mice with leptin supplementation during severe influenza A infection. (A) Fasting glucose of mice. (B) Weight of mice. Baseline (C) heart rate and (D) core temperature of control ob/ob mice (Ob-Ctrl) and ob/ob mice supplemented with 75 ng/hr of leptin (Ob-Leptin). (E) Daily food consumption of mice over the course of infection. (F) Mouse body weight over course of infection. (G) Cumulative weight loss of mice over the course of infection from (F). (H) Probability of survival with acute leptin supplementation. (I) Daily food consumption and (J) body weight during acute leptin supplementation. DPI, days post infection. Other abbreviations as per figure 1. *, p<.05; **, p <.01; ***, p<.005 per Student's T-test with correction for multiple comparisons; #, only surviving mice were used to plot graphs.

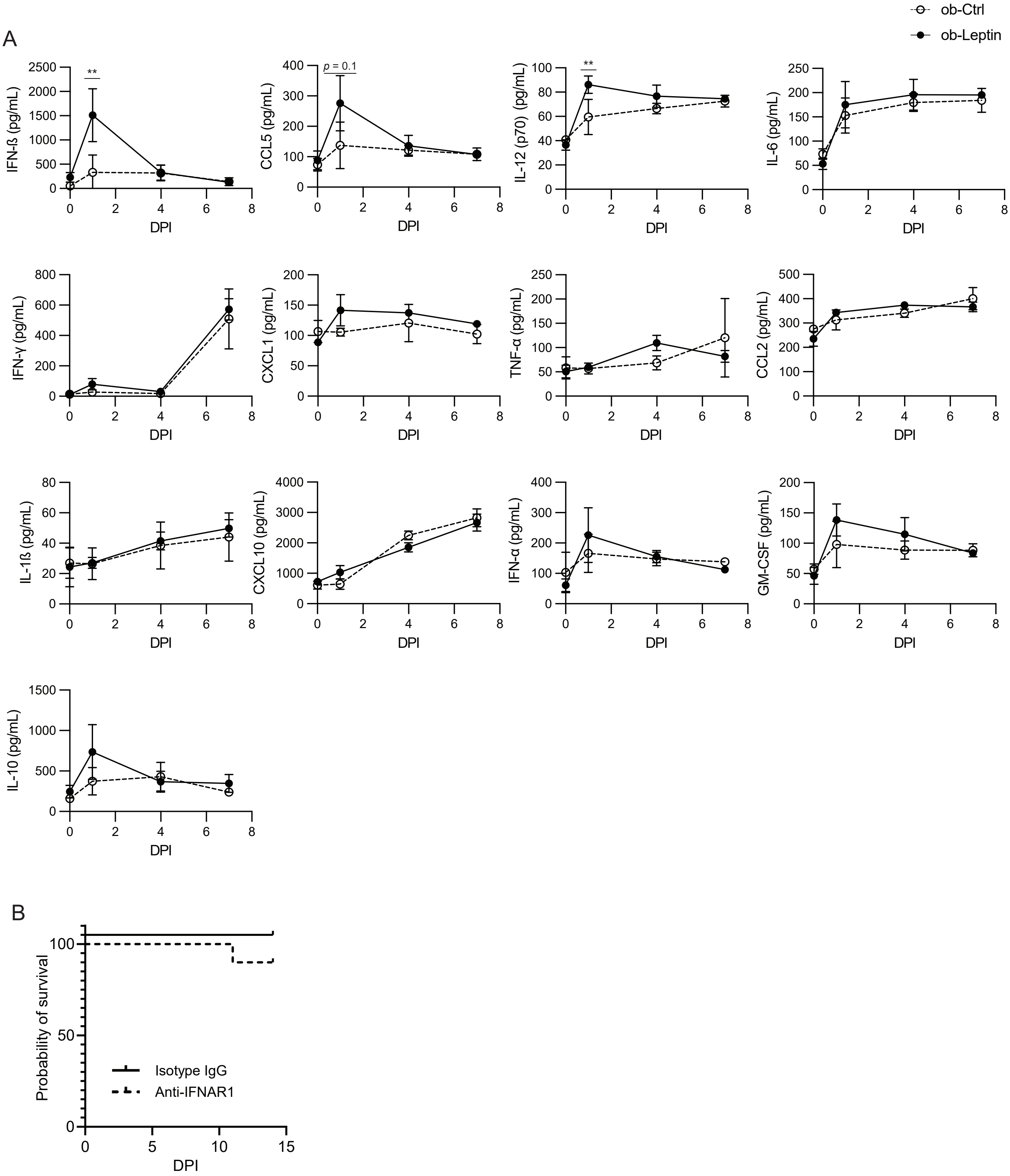

Figure S2. Cytokine signaling and leptin supplementation. (A) Cytokine response to leptin treatement. (B) Survival with blockade of IFNAR1 in mice supplemented with 75 ng/hr of leptin. **, p<.01. Other abbreviations as per Figure 1.

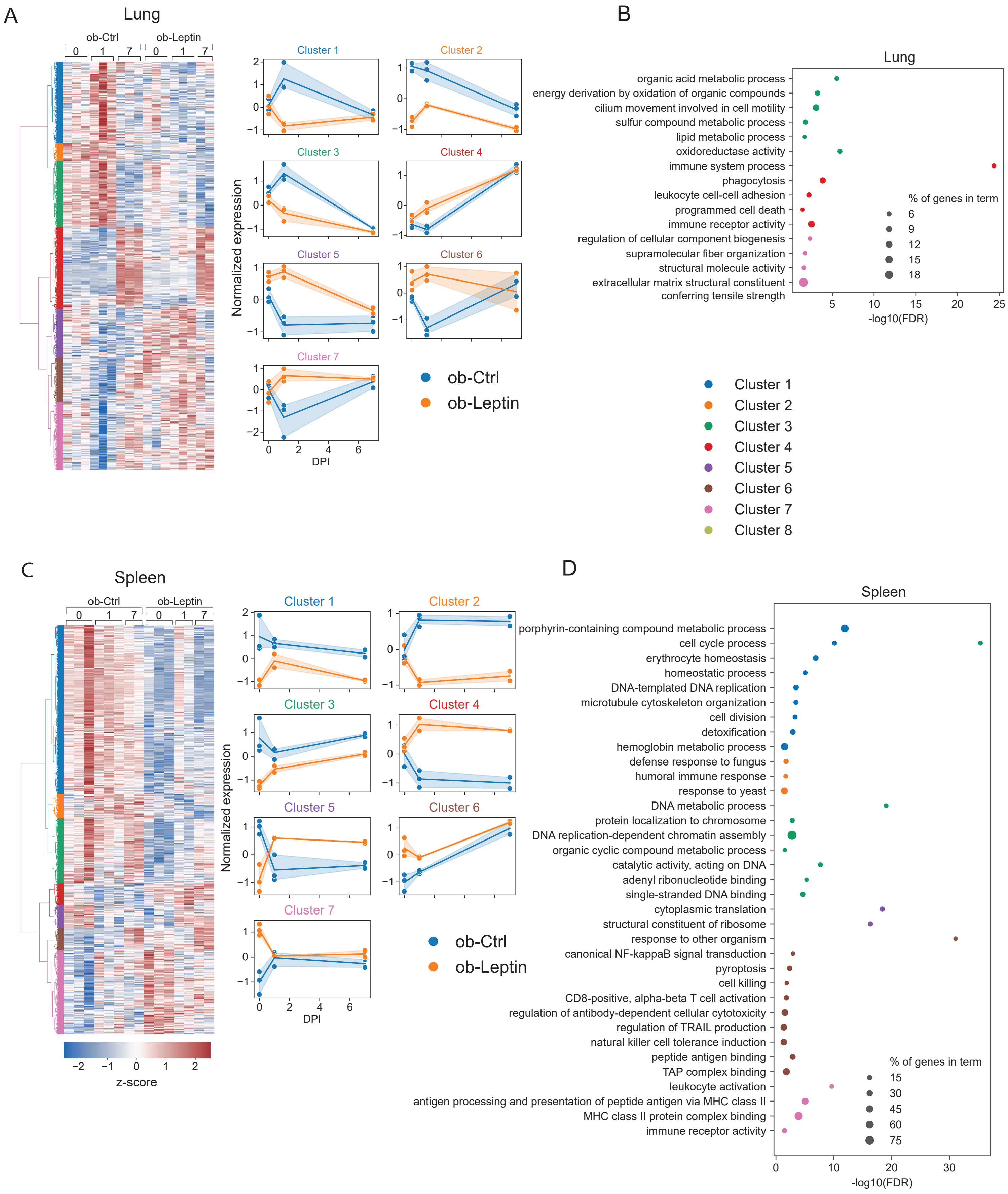

Figure S3. Whole tissue RNAseq gene clustering analysis. Clustered heatmap and mean expression over time for lung (A) and spleen (C). Gene set enrichment analysis of lung (B) and spleen (D) gene clusters. FDR, false discovery rate. Other abbreviations as per Figure 1.

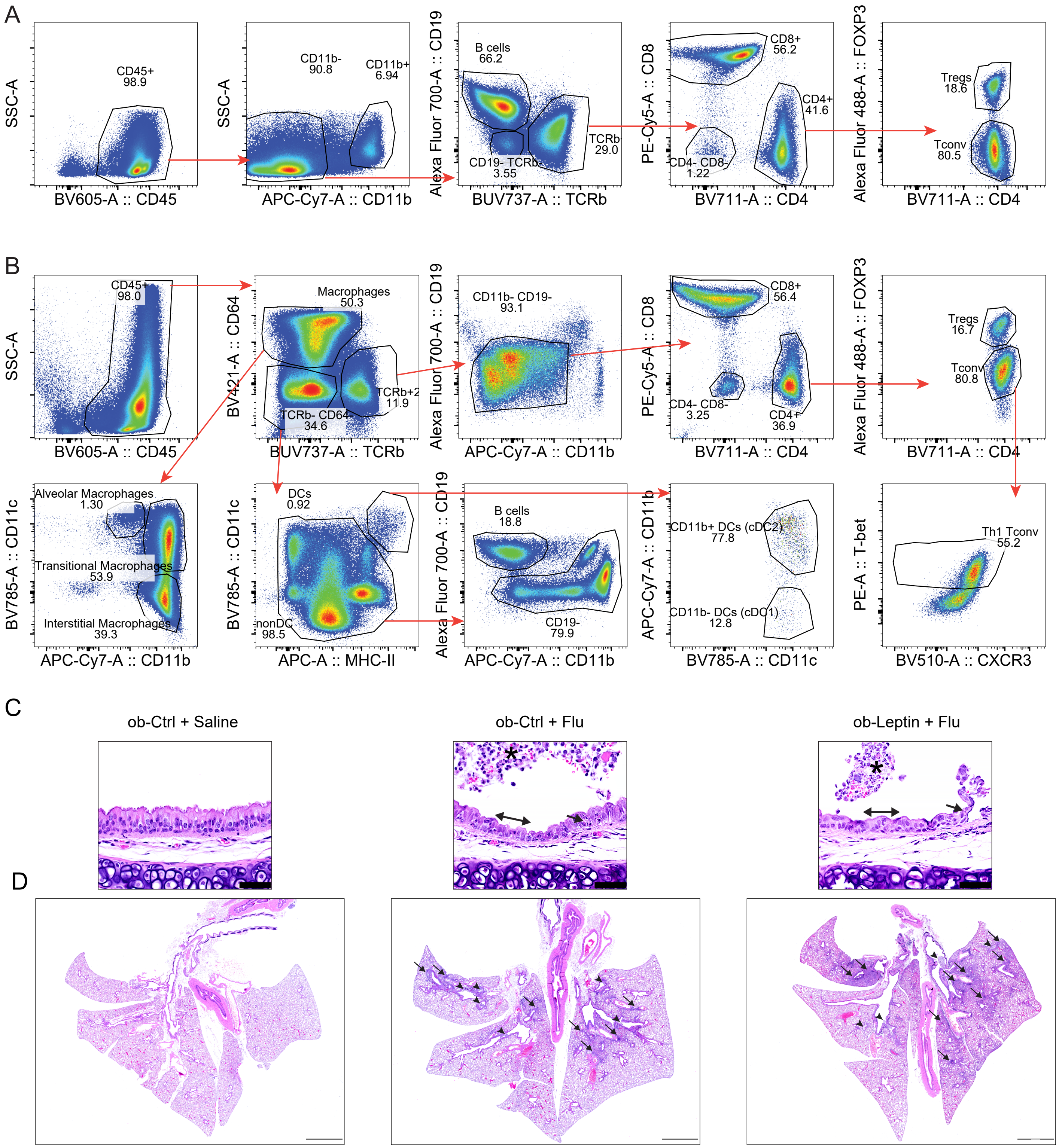

Figure S4. Gating strategy for flow cytometry analysis of spleen (A) and lung (B). (C) H&E staining of trachea from *ob/ob* mice. The uninfected tissue (left) shows a normal trachea lined by pseudostratified columnar epithelial cells. Seven days post infection, both control infected (middle) and leptin infected (right) tissues show attenuation of the epithelium (black double-headed arrows), necrotic epithelial cells (black arrows), and luminal exudates with cellular and karyorrhectic debris (black asterisk) with magnification using a 20x objective and scale bar 40µM. (D) Corresponding H&E staining of whole lung with hypercellularity and parencymal consolidation (arrows) and bronchiolar luminal exudates with necrotic debris (arrowheads) with magnification using a 1x objective and scale bar 2 mM. MHC, major histocompatibility complex. Other abbreviations as per Figure 1.
